# Spatial-temporal analysis of climate and socioeconomic conditions on cholera incidence in Mozambique from 2000 to 2018: an ecological longitudinal retrospective study

**DOI:** 10.1136/bmjopen-2023-082503

**Published:** 2024-08-19

**Authors:** Chaibo Jose Armando, Joacim Rocklöv, Mohsin Sidat, Yesim Tozan, Alberto Francisco Mavume, Aditi Bunker, Maquins Odhiambo Sewe

**Affiliations:** 1 Department of Public Health and Clinical Medicine, Sustainable Health Section, Umeå University, Umea, Sweden; 2 Eduardo Mondlane University, Maputo, Mozambique; 3 Heidelberg Institute of Global Health and Interdisciplinary Centre for Scientific Computing, Heidelberg University, Heidelberg, Germany; 4 School of Global Public Health, NYU, New York, New York, USA; 5 Heidelberg Institute of Global Health, Heidelberg University, Heidelberg, Germany; 6 Universitatsklinikum Heidelberg Heidelberg Institute of Global Health, Heidelberg, Germany

**Keywords:** Epidemiology, Infection control, Public health, Risk Factors

## Abstract

**Abstract:**

**Objectives:**

This study aims to assess both socioeconomic and climatic factors of cholera morbidity in Mozambique considering both spatial and temporal dimensions.

**Design:**

An ecological longitudinal retrospective study using monthly provincial cholera cases from Mozambican Ministry of Health between 2000 and 2018. The cholera cases were linked to socioeconomic data from Mozambique Demographic and Health Surveys conducted in the period 2000–2018 and climatic data; relative humidity (RH), mean temperature, precipitation and Normalised Difference Vegetation Index (NDVI). A negative binomial regression model in a Bayesian framework was used to model cholera incidence while adjusting for the spatiotemporal covariance, lagged effect of environmental factors and the socioeconomic indicators.

**Setting:**

Eleven provinces in Mozambique.

**Results:**

Over the 19-year period, a total of 153 941 cholera cases were notified to the surveillance system in Mozambique. Risk of cholera increased with higher monthly mean temperatures above 24°C in comparison to the reference mean temperature of 23°C. At mean temperature of 19°C, cholera risk was higher at a lag of 5–6 months. At a shorter lag of 1 month, precipitation of 223.3 mm resulted in an 57% increase in cholera risk (relative risk, RR 1.57 (95% CI 1.06 to 2.31)). Cholera risk was greatest at 3 lag months with monthly NDVI of 0.137 (RR 1.220 (95% CI 1.042 to 1.430)), compared with the reference value of 0.2. At an RH of 54%, cholera RR was increased by 62% (RR 1.620 (95% CI 1.124 to 2.342)) at a lag of 4 months. We found that ownership of radio RR 0.29, (95% CI 0.109 to 0.776) and mobile phones RR 0.262 (95% CI 0.097 to 0.711) were significantly associated with low cholera risk.

**Conclusion:**

The derived lagged patterns can provide appropriate lead times in a climate-driven cholera early warning system that could contribute to the prevention and management of outbreaks.

**STRENGTHS AND LIMITATIONS OF THIS STUDY**

- Employs an ecological longitudinal retrospective design to analyse temporal and spatial trends in cholera incidence, yielding insights into the dynamics.
- The method comprehensively elucidates cholera incidence in Mozambique by exploring the influence of climate and socioeconomic conditions employing advanced statistical techniques to explore the complex relationships.
- Understanding the lagged effects of climate and socioeconomic factors is essential for discerning the temporal delay between risk exposure and cholera incidence, offering insights into long-term dynamics for effective management.
- The use of cross-sectional Demographic and Health Survey data, collected every 5 years and aggregation to provincial, may have masked the relationship with cholera.

## Introduction

 Cholera is a waterborne disease caused by the bacteria *Vibrio cholerae* with the whole population at risk. Humans infection of cholera mostly occurs through faecal-oral route by the ingestion of contaminated food or water.^1^,^3^ Improved access to water, sanitation and hygiene (WASH) has been shown to decrease cholera occurrence by interrupting transmission routes.^4^,^7^ However, cholera continues to affect vulnerable populations in low-resource settings with inadequate water and sanitation infrastructure.^8^,^10^ Cholera imposes a substantial global burden, with an annual estimated incidence of 1.3–4 million cases worldwide and results in high mortality rates, ranging from 21 000 to 143 000 deaths each year.^411^,^13^ Globally, the number of cholera cases showed a significant decline from 2014 to 2017. In 2015, there was a 9.49% reduction in reported cases compared with the 190 549 cases in 2014. This downward trend continued with a further 30.66% decrease in 2016, followed by an additional 37.02% decline in 2017. These figures indicate a consistent and substantial reduction in cholera cases worldwide over this period.^11 14^ Cholera remains endemic in sub-Saharan Africa (SSA)^15^ and more than 87 million people live in areas with high risk of cholera transmission.^16^ Between 1970 and 2011, there were a total of 3 221 050 cholera cases, and 9.79% (315 295) of the cases were reported in Mozambique.^17^ Between 2000 and 2015, 83% of the cholera mortalities reported by the WHO occurred in SSA.^18^ In 2017, there were 3220 deaths among the reported 1 79 835 cases, a case fatality rate of 1.8%.^19^ SSA including Mozambique experiences year-round transmission of cholera.^19 20^ Mozambique registered the first cholera cases in early 1973.^21 22^ Notified cholera cases from Mozambique account for about one-fifth to one-third of all cases reported in Africa across the years.^21 23^ In 2019, Sofala Province in Mozambique reported 6766 cholera cases with 8 associated fatalities.^24^ According to the United Nations Office for the Coordination of Humanitarian Affairs report, Mozambique recorded approximately 26 841 cholera cases and 123 fatalities in April 2023.^25^

The ongoing cholera burden in Mozambique is attributed to inadequate sanitation and extreme weather events, such as tropical cyclones and droughts^26^,^29^ though the proportion of the population who have access to adequate sanitation in Mozambique has increased from 28% in 2015^27^ to 38% in 2022.^30^ Studies have investigated how WASH improvements can reduce the risk of cholera transmission in Mozambique,^31 32^ Congo,^33^ Niger^34^ and Kenya^35^ and yet the effectiveness of these interventions has been not established.^36^ The key association between cholera risk and WASH factors can help to identify and prioritise areas with the highest need for intervention.^37^,^39^ In addition to the WASH factors, cholera transmission is affected by various climatic factors such as temperature and precipitation.^40^,^42^ Temperature is considered as an important factor for *V. cholerae* occurrence and propagation in the water reserves such as river, lake, oceans, stagnated water and groundwater.^43 44^ Furthermore, precipitation influences cholera transmission by increasing the risk of water contamination and the concentration of *V. cholerae* through runoff.^45 46^ These climate factors affect the temporal and spatial range of the *V. cholerae* and influence exposure pathways.^47^ In Mozambique, cholera transmission generally peaks between December and April during the hot and rainy season.^48 49^

Mozambique is a low-income country with weak WASH infrastructure and limited resources to scale up interventions against cholera outbreaks. Cholera has been linked to the households with low socioeconomic conditions, poverty, inequalities, education level and poor sanitation.^31 50^ The incubation period of cholera is relatively short ranging from just 2 hours to 5 days, thus the cases can increase quite substantially in a relatively short time.^51 52^ Identification of vulnerable areas for multisectoral interventions can be a great first step for cholera control and mitigation.^53^ Several interventions are used for cholera control and prevention including distribution of oral cholera vaccine, improved drinking water and sanitation facilities for communities at risk areas, as well as provisions of adequate healthcare.^54 55^ In this study, we aim to assess how climate and socioeconomic conditions affect cholera incidence in Mozambique considering the lagged patterns over space and time. Through analysis of temporal lag patterns, we can better understand and respond to the dynamics of cholera transmission, leading to more effective prevention and control strategies.

## Materials and methods

### Study area

Mozambique is one of the most vulnerable African countries to climate change along its coasts,^56^ with the majority of people living in rural areas along the coast.^57^ Mozambique is bounded by Tanzania on the north, Malawi and Zambia on the north-west, Zimbabwe on the west, South Africa and Swaziland on the south-west and the Indian Ocean on the east (see online supplemental figure S1).

### Data

Weekly cholera cases at the province level were extracted from the Mozambique Ministry of Health disease surveillance system for the years 2000–2018 and aggregated to monthly totals. Data on the climatic factors, including daily precipitation, relative humidity (RH), Normalised Difference Vegetation Index (NDVI), and minimum and maximum temperature ($T_{min}$, $T_{max}$), were sourced from National Center for Environmental Prediction (NCEP).^58^

The NCEP employs advanced computer models that integrate observational data to simulate and predict weather patterns, including temperature, precipitation, NDVI and RH. These diverse data collection methods enable NCEP to generate reliable and detailed climate data which is available globally at different resolutions.^59^ Monthly summaries of the daily climatic data were computed and summarised at the provincial level based on shapefiles.^60^ In addition, the population data used in the analysis was sourced from Woldpop.^61^

Socioeconomic data from the Demographic Health Surveys (DHS) for the years 2003, 2009 and 2015 were included in the analysis.^62^ The socioeconomic variables included in this study followed similar variables used in Armando *et al*^63^ and were aggregated to the provincial level by applying the method used in Armando *et al*^63^ (see online supplemental tables S1 and S2).

### Patient and public involvement

No patients or the public were involved in the design, conduct, reporting or dissemination of this study.

### Statistical analysis

We employed an ecological longitudinal retrospective study design to analyse the temporal and spatial pattern of cholera incidence rates in Mozambique. We applied distributed lag non-linear models (DLNM)^64^ in a Bayesian framework with integrated nested Laplace approximation (INLA)^65^ to model the delayed and non-linear relationship between minimum temperature ($T_{min}$), mean temperature ($T_{mean}$), maximum temperature ($T_{max}$), total precipitation, RH and NDVI and cholera incidence adjusting for the DHS-derived socioeconomic indicators. In DLNM methodology, a bidimensional cross-basis function is created in order to simultaneously capture the lag and the variable dimension spaces.^64^ A natural cubic spline with 3 df was used for both the lag and the variable dimension. Lags of 0–6 months for climatic variables were assessed. The overdispersed cholera cases were modelled using a negative binomial distribution. Backward elimination based on significance at a 95% credible interval was used to select the socioeconomic variables in the final model.

A significance level of 5% means that there is a 5% chance of incorrectly rejecting the null hypothesis. The final model consisted of the cross-basis functions of the climatic variables and the significant socioeconomic factors.

We used the following reference values 18°C, 23°C, 28°C, 77 mm, 73%, 0.2 for $T_{min}$, $T_{mean}$, $T_{max}$, precipitation, RH and NDVI, respectively, when defining cross-basis functions. Interpretations are made in reference to these values.

The final model selected is represented in equation (1) below:

$$Y_{it}∼Negbin$$

$$log(Y_{it)}=\beta_{o}+\left(u_{i}+\nu_{i}\right)+\omega_{t}+f\left(x_{j},vardf,lagdf\right)+log(E_{t})\left(1\right)$$

$Y_{it}$ represents cholera incidence rate for province i in month t, $\beta_{o}$ is the intercept, $u_{i}$ is the provincial exchangeable area effect while $\nu_{i}$ is the spatially structured random area effect. $\omega_{t}$ is the monthly random effect modelled with random walk of order 1. $f\left(x_{j},vardf,lagdf\right)$ represents the defined cross-basis function for climate and socioeconomic variable $x_{j}$ with vardf and lagdf representing the df for the variable and lag basis function. $E_{t}$ is the population offset added to the model with a coefficient of 1.

We used default INLA prior specifications for the provincial and monthly random effects.

All the analyses were done using R V.4.2.0^66^ while DLNM^64^ and INLA^65^ packages in R were used for the analysis.

## Results

### Cholera cases and environmental variables

There were 153 941 cholera cases reported between 2000 and 2018 in the whole of Mozambique. The annual cholera incidence rates ranged from a high of 181.5 in the year 2002 to a low of 1.8 cases per 100 000 population in the year 2014 (see online supplemental tables S3 and S4). In the periods 2005–2009, 2010–2014 and 2015–2018, Mozambique experienced huge reductions in cholera incidence rates. For example, between 2005 and 2009, cholera incidence rate was 531.2 per 100 000 population with a decrease by 68.91% when considering the period 2000–2004 when it was 167.3 per 100 000 population. This downward trend continued in 2010–2014, with a further reduction of 92.54% to a rate of 40.1 per 100 000. By 2015–2018, cholera incidence rate had decreased by 92.74% compared with the 2000–2004 period to 39.1 per 100 000 (table 1).

**Table 1:** Summary of monthly cholera cases and environmental factors in Mozambique from 2000 to 2018

	2000–2018	2000–2004	2005–2009	2010–2014	2015–2018
Cholera cases					
Min	0	11	0	0	0
Max	8349	8349	5579	1529	1750
Mean (SD)	675.18 (1408.68)	1612.95 (2203.69)	613.42 (1099.62)	160.68 (305.78)	223.29 (422.78)
Cholera incidence rate per 100 000					
Min	0	0.61	0	0	0
Max	494.70	494.70	270.73	72.17	71.85
Mean (SD)	37.00 (81.12)	95.38 (130.24)	30.67 (53.65)	7.37 (14.17)	8.96 (17.26)
Minimum temperature					
Min	13.32	14.09	13.96	13.32	14.50
Max	23.0	22.75	23.0	22.69	22.76
Mean (SD)	18.98 (2.94)	19.14 (2.92)	19.11 (3.00)	18.70 (3.03)	18.97 (2.83)
Mean temperature					
Min	18.65	18.98	19.20	18.65	19.59
Max	27.34	26.84	27.34	26.99	27.34
Mean (SD)	23.73 (2.44)	23.64 (2.46)	23.98 (2.45)	23.47 (2.50)	23.85 (2.37)
Maximum temperature					
Min	23.65	23.66	24.36	23.65	24.59
Max	31.92	31.30	31.74	31.56	31.92
Mean (SD)	28.48 (2.06)	28.14 (2.11)	28.85 (2.00)	28.24 (2.06)	28.74 (2.01)
Normalised Different Vegetation Index					
Min	0.121	0.121	0.171	0.165	0.129
Max	0.365	0.365	0.343	0.349	0.331
Mean (SD)	0.240 (0.050)	0.240 (0.050)	0.250 (0.060)	0.240 (0.050)	0.230 (0.050)
Relative humidity					
Min	59.17	60.41	59.79	59.38	59.17
Max	91.48	91.48	89.79	89.52	88.62
Mean (SD)	73.88 (9.19)	74.86 (9.26)	72.75 (9.18)	74.15 (9.35)	73.72 (9.03)
Precipitation					
Min	1.35	2.42	2.48	1.35	2.89
Max	345.01	345.01	262.25	344.84	260.31
Mean (SD)	77.60 (80.85)	90.28 (90.52)	74.68 (79.34)	73.79 (78.48)	70.18 (73.20)

**Figure 1:**
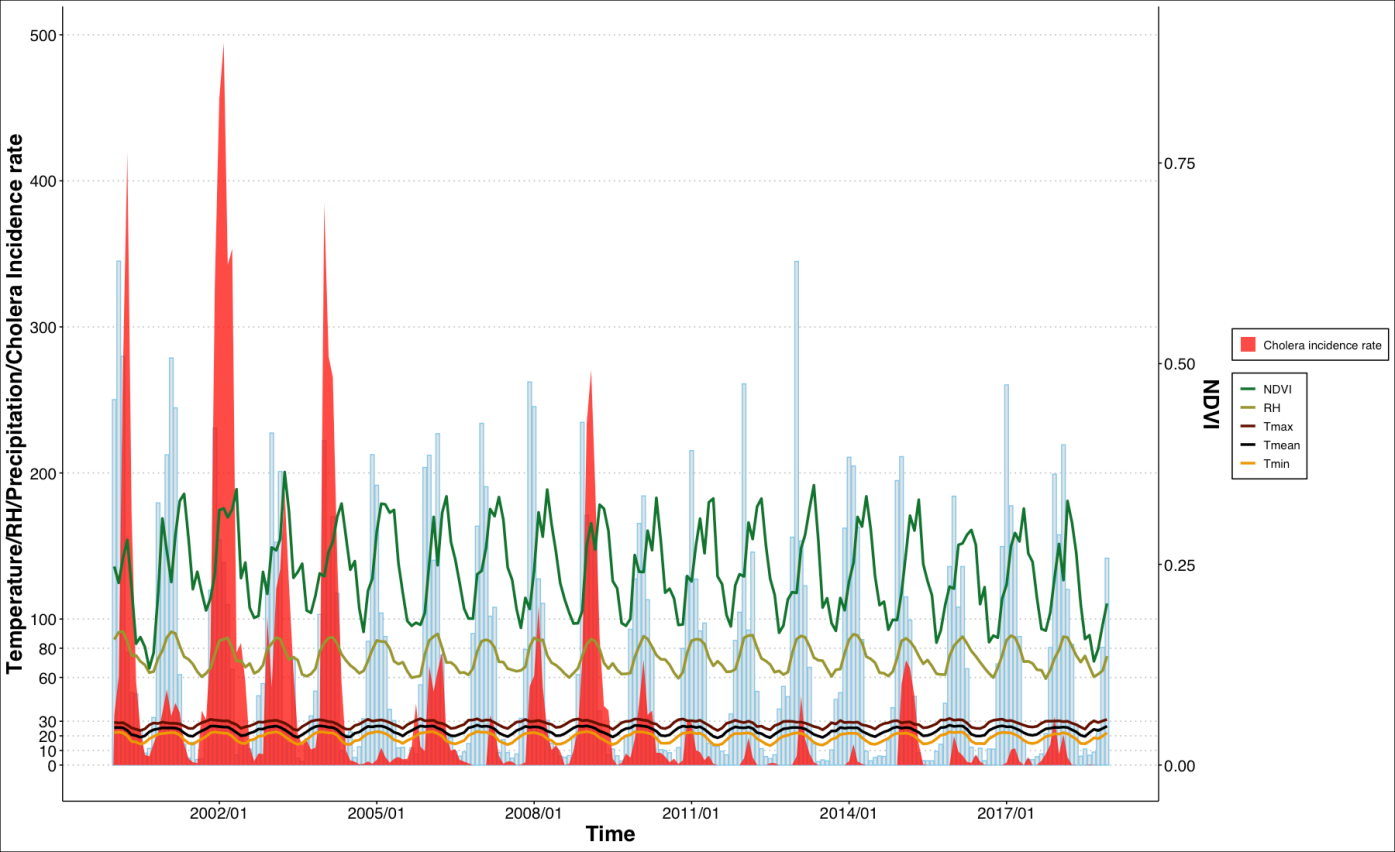
Seasonal variation in the monthly cholera incidence rates (red) and monthly precipitation (light blue), minimum temperature (orange), mean temperature (black), maximum temperature (dark red), relative humidity (light green) and NDVI (green) in Mozambique from 2000 to 2018. NDVI, Normalised Difference Vegetation Index.

The seasonal variation in cholera incidence rates followed the seasonal variation in environmental factors (figure 1, see online supplemental figure S2). During the period 2000–2018, cholera incidence rate decreased, and cholera burden was geographically concentrated in the central and northern parts of Mozambique (figure 1, see online supplemental figure S3-C and S4). The year 2014 had the lowest national average of 40 cholera cases per month while the year 2002 had the highest mean of about 2809 cases, as shown in online supplemental table S4. Cholera outbreaks in Mozambique show a strong seasonal pattern with an increased burden from November to April (figure 1). Cholera seasonality in Mozambique varies geographically, with peak transmission occurring earlier in the year in the northern regions, such as Cabo Delgado and Nampula provinces, and central regions, including Zambezia, Sofala and Manica provinces (see online supplemental figure S4). Over the study period, the northern and central regions of Mozambique received more precipitation compared with other parts of the country. The driest provinces were Maputo, Gaza and Maputo City (see online supplemental figure S5). The highest temperatures (minimum, mean and maximum) are observed along the central and northern coast while the lowest temperatures occur in Gaza and Maputo province (see online supplemental figure S6). Mozambique exhibits a simple seasonal temperature profile, with the lowest temperatures in July and the highest in December. Between 2000 and 2018, the annual mean RH in Mozambique ranged from 63.6% to 79.8% (see online supplemental figure S9), indicating significant fluctuations in atmospheric moisture levels over the years. Lower NDVI values are seen in Maputo City, Gaza, Maputo, Tete and Niassa provinces (see online supplemental figure S10) while the higher NDVI values are found in the central provinces such as Sofala, Manica and Zambezia (see online supplemental figures S10 and S11).

### Socioeconomic indicators

Summaries of the DHS-derived socioeconomic and WASH factors aggregated at the provincial level are shown in online supplemental table S1.

Specifically considering access to clean water and adequate sanitation, two key promoters of overall health and well-being, the majority of households (56.8%) in the provinces reported no access to treated drinking water (see online supplemental table S1). The proportion of households that shared a toilet facility with other households differed across the provinces ranging from 4% to 17% with an average of 10.1%. The average proportion of households with radio and mobile phone ownership was 39%, 67% with a range of 27%–55% and 45%–97%, respectively. As for educational attainment, on average, 25.1% of households in the provinces reported no education while the highest was 38.6% (see online supplemental table S1).

### Model results

#### Temperature

**Figure 2:**
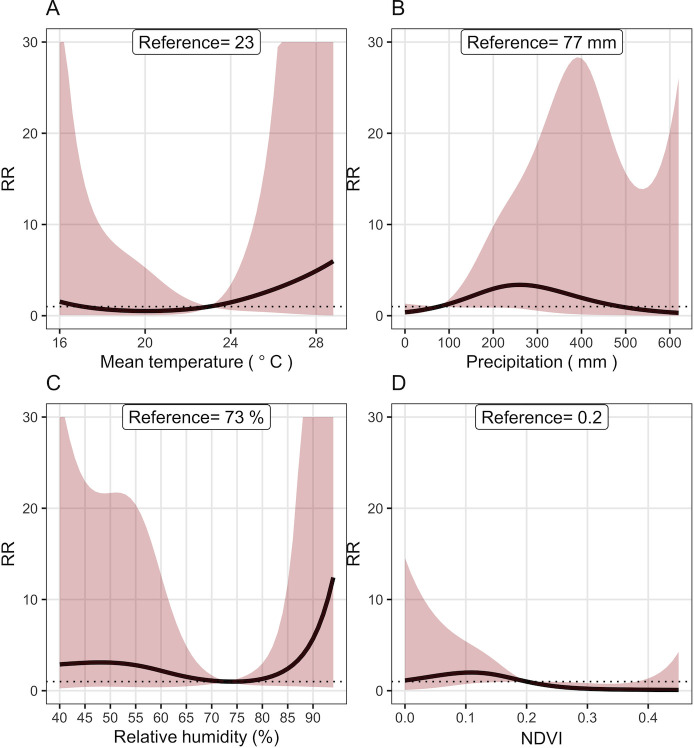
Overall effect of (**A**), precipitation (**B**), relative humidity (**C**) and NDVI (**D**) on cholera risk in Mozambique, 2000–2018. The reference values for, precipitation, relative humidity and NDVI were 23°C, 77 mm, 73% and 0.2, respectively. NDVI, Normalised Difference Vegetation Index.

Table 2 displays the relationship between mean temperature and cholera risk at different lags and percentiles. The relative risk (RR) of cholera gradually increased as minimum, mean and maximum temperature increased (figure 2A, see online supplemental figure S12). We observed a lower risk of cholera for mean temperatures between 19°C and 22°C at lags 0–4 months, compared with the reference of 23°C. The highest RR of cholera was at 28°C with a lag of 0 months (table 2). At 19°C, cholera risk was also high compared with that the reference but with a much longer lag of 5–6 months. Lower risk of cholera was, however, observed at much shorter lags. We observed an increasing RR of cholera for a mean temperature above 24°C (table 2). We observed an increased risk of cholera at maximum temperatures between 24°C and 33°C with lags of 2–3 months, compared with the reference value of 28°C, although this finding was not statistically significant (see online supplemental table S5).

**Table 2:** The association between mean temperature, precipitation, relative humidity, NDVI and cholera risk at lags 0–6 months

Lag	Mean temperature (Ref=23°C)						
	Percentile						
	5th	10th	25th	50th	75th	90th	99th
	19	20	22	24	26	27	28
0	0.72	0.70	0.85	1.15	1.28	1.24	1.54
1	0.79	0.78	0.90	1.11	1.22	1.21	1.19
2	0.87	0.87	0.93	1.10	1.17	1.20	1.22
3	0.94	0.94	0.97	1.04	1.14	1.19	1.25
4	1.00	1.00	0.99	1.02	1.12	1.20	1.29
5	1.07	1.05	1.00	1.01	1.11	1.21	1.33
6	1.13	1.10	1.02	1.00	1.12	1.23	1.37
	Precipitation (Ref=77 mm)						
	1.2	2.4	7.4	33.7	114.5	223.3	395.1
0	0.75	0.76	0.77	0.85	1.14	1.57*	1.80
1	0.78	0.78	0.79	0.87	1.22*	1.46*	1.53
2	0.86	0.86	0.87	0.92	1.07	1.21	1.12
3	0.99	0.99	0.99	0.99	1.00	0.95	0.81
4	1.05	1.05	1.05	1.03	0.97	0.88	0.77
5	0.96	0.96	0.96	0.98	1.02	1.02	0.91
6	0.76	0.77	0.78	0.86	1.12	1.37	1.15
	Relative humidity (Ref=73%)						
	54	61	67	74	82	88	92
0	0.56	0.61	0.78	1.03	1.13	1.00	0.89
1	0.85	0.87	0.92	1.01	1.08	1.09	1.09
2	1.22	1.16	1.07	0.99	1.04	1.18	1.32
3	1.53*	1.40*	1.17	0.98	1.03	1.26	1.50
4	1.62*	1.45*	1.19*	0.98	1.04	1.31	1.62
5	1.51*	1.36	1.15	0.98	1.07	1.35	1.65
6	1.32	1.20	1.08	0.99	1.11	1.37	1.64
	NDVI (Ref=0.2)						
	0.137	0.157	0.189	0.238	0.295	0.337	0.400
0	1.02	1.00	1.00	0.99	1.00	1.03	1.09
1	1.12	1.09	1.03	0.91	0.81	0.79	0.83
2	1.20*	1.15*	1.04*	0.85*	0.69	0.65*	0.67
3	1.22*	1.17*	1.05*	0.83*	0.65*	0.59*	0.59*
4	1.16*	1.13*	1.03*	0.87*	0.70*	0.63*	0.59*
5	1.05	1.04	1.01	0.94	0.84	0.76	0.65
6	0.93	0.94	0.98	1.05	1.06	0.97	0.74

* Significant.

NDVINormalised Difference Vegetation Index

#### Precipitation

Table 2 shows the estimated relationship between precipitation and relative cholera risk at lags 0–6 months. There were delayed effects on cholera risk with precipitation, with the maximum effects of heavy precipitation reached at shorter lags of less than 1 month. For example, total precipitation of 223.3 mm with a lag of 1 month, a resulted in 57% increase in cholera risk (RR 1.57 (95% CI 1.06 to 2.31)). We also observed that the effect of heavy precipitation on cholera risk was attenuated at lags of 3–5 months. The overall association between cumulative precipitation and cholera risk is displayed in figure 2B. Increasing amounts of precipitation were associated with increased cholera risk at lags 0–2 months (table 2). The RR of cholera decreased as precipitation increased at a lag of 4 months (table 2). At precipitation of above 500 mm, cholera risk was lower (figure 2B).

#### Relative humidity

Table 2 shows exposure-lag response surface for RH and cholera risk. For RH between 54% and 67%, the risk of cholera increased at longer lags for RH of 54% (RR 1.620 (95% CI 1.124 to 2.342)) at a lag of 4 months. This association is more intense at a lag of 3–5 months. Higher RH values were found to be significantly associated with cholera risk at longer lags. At an RH of 67%, cholera risk increased by 19% (RR 1.190 (95% CI 1.017 to 1.392) at a lag of 4 months. The non-linear relationship between RH and cholera risk is shown in figure 2C. For RH greater than 80% and below 70%, cholera risk consistently increased though the association was not statistically significant (figure 2C).

#### Normalised Difference Vegetation Index

Table 2 displays the lag–response association between NDVI and cholera risk. For NDVI of 0.137, cholera risk was highest at a lag of 3 months (RR 1.220 (95% CI 1.042 to 1.430)). We observed a decreased RR for NDVI values from 0.238 to 0.400 compared with the reference value of 0.2, for example, at an NDVI value of 0.337 cholera risk was 37% lower (RR 0.630 (95% CI 0.468 to 0.857)), compared with the reference. The overall relationship between NDVI and cholera risk is displayed in figure 2D. In comparison to the reference value of 0.2, cholera risk was significantly lower for NDVI values above 0.22 (figure 2D). Specifically, at a monthly mean NDVI of 0.38, cholera risk was 88.9% lower, (RR 0.110 (95% CI 0.012 to 0.984)), and at 0.27, it was 66.78% lower (RR 0.332 (95% CI 0.118 to 0.928)) (figure 2D). For NDVI values below the reference value of 0.2, the risk of cholera slightly increased though it did not show a significant relationship with cholera risk (figure 2D).

#### Socioeconomic factors for cholera

Table 3 shows the association between household socioeconomic indicators and cholera risk. A high proportion of households with a radio (used for delivering outbreak messages) was significantly associated with very low risk, 70.1% lower (RR 0.29 (95% CI 0.109 to 0.776)), compared with having a low percentage of radio ownership. Similarly, high proportion of mobile phones ownership was associated with a 73.76% decreased risk of cholera compared households with low ownership (RR 0.262 (95% CI 0.097 to 0.711)). Conversely, we observed an increased risk of cholera in households sharing a toilet, though this was not statistically significant.

**Table 3:** The association between socioeconomic variables and cholera risk

	Radio ownership (Ref=26.68%)					
	Percentile					
	5th	10th	25th	50th	75th	90th
	27	37	39	40	41	50
RR	1.01	0.56	0.39	0.33*	0.28*	0.29*
	**Mobile phone ownership (Ref=45%)**					
	**45.4**	**45.7**	**46.9**	**69.9**	**86.1**	**87.9**
RR	0.997	0.994	0.986	0.697	0.262*	0.292*
	**Toilet sharing (Ref=7%)**					
	**4.02**	**5.02**	**7.76**	**9.23**	**13.13**	**14.2**
RR	1.096	1.034	1.043	1.212	1.302	1.108

The reference value from households with a radio, mobile phones and those who share toilet facilities is 26.68%, 45% and 7%, respectively

* Significant.

RRrelative risk

## Discussion

We analysed the non-linear relationship between delayed climatic conditions and cholera risk in Mozambique while adjusting for socioeconomic conditions, space and time dependencies in a Bayesian framework making inferences using the computationally efficient INLA methodology. We show that temperature, precipitation, RH and NDVI influence the spatiotemporal distribution of cholera incidences in Mozambique.

Meteorological factors play a crucial role in cholera transmission pathways, for example, high temperatures in a warmer season may provide a suitable condition for *V. cholerae* to proliferate.^42^,^4467^ In this study, we found that mean temperature above 24°C increased cholera risk at lags 0–6 months. At mean temperatures between 19°C and 22°C, the risk was lower at shorter lags. A study looking at countries in SSA linked elevated cholera risk with mean temperature at lags above 2 months^1^ which is consistent with our study. In Zanzibar, cholera risk increased with 1°C rise in temperature with a delay of 4 months.^68^ In Tanzania, they also found a significant relationship between temperature and cholera risk, however, they did not consider the lagged effect of temperature.^69^

Similar to temperature, precipitation also modulates cholera risk acting at different spatial and temporal scales. In this study, we found that increased precipitation was associated with an increased risk of cholera occurrence at a 1-month lag. With monthly precipitation of between 150 and 500 mm, the risk of cholera was high at a shorter lag of 1 month and reduced at higher lags of 3–5 months. This resonates with our finding as the incubation period for *V. cholerae* causing cholera, is usually less than a month with most symptoms appearing within 1–3 days after ingestion of contaminated food or water. In South Sudan, precipitation was found to be the most important driver for cholera, however, this study also did not explore the delayed effect of precipitation.^45^ In Bangladesh, a significant 1-month delay, similar to our finding, was found between precipitation and cholera risk where a 1 mm/day increase in mean precipitation was linked with a 6.5% increase in cholera transmission,^70^ while in this study, we found increase of 57% at precipitation of 223.3. In Zambia, higher cholera risk was found at a lag less than 1 month for precipitation above 50 mm.^71^ Though in South Africa,^72^ precipitation had the strongest association with cholera risk at 2-month lag, while in Haiti,^73^ shorter lags less than 1 month were found, very similar to our findings.

We found a significant association between NDVI values and cholera risk, with lower NDVI values linked to higher cholera risk and higher NDVI values associated with reduced risk. Lower NDVI values are often associated with dry or drought conditions, which can exacerbate cholera transmission. During droughts, water scarcity forces populations to rely on contaminated water sources, such as stagnant ponds, rivers and lakes for drinking and household purposes, thereby increasing the risk of cholera transmission. NDVI is the most used vegetation index in different areas including epidemiology and climate variability.^74^ NDVI increases with the amount of green biomass and precipitation. In this study, we found NDVI to be positively associated with precipitation.

Our findings indicated that RH levels between 54% and 67% were linked to an increased risk of cholera, with effects observed after delays ranging from 0 to 6 months. Specifically, the risk of cholera was notably higher when longer lag periods were considered within this RH range.

This is consistent with the study done in India.^75^ Similarly, in Zanzibar, cholera outbreaks were found to be significantly associated with RH at a lag of 5 months.^68^ RH critically influences cholera transmission dynamics by modulating the survival of *V. cholerae* bacteria in environmental reservoirs and impacting human susceptibility to infection. RH directly influences the persistence of *V. cholerae* in various water sources, including rivers, ponds and coastal areas, potentially extending the bacterium’s survival duration in water and thereby augmenting the risk of exposure through contaminated water sources.

Household’s socioeconomic disparities can help elucidate the fluctuations in cholera transmission between and within provinces in Mozambique during the outbreak in addition to the climatic factors. Studies done in Zambia^76 77^ and Kenya^78 79^ showed that households sharing toilet facilities were at higher risk of cholera incidences, a finding consistent with our study. Sharing toilet facilities increases cholera risk due to factors such as inadequate sanitation, increased cross-contamination and limited access to hygiene resources. Shared toilet facilities often lack proper sanitation and maintenance, resulting in the accumulation of faecal matter and increased likelihood of faecal-oral transmission of *V. cholerae*.

A study conducted in Haiti found that household with radio had lower cholera risk^80^ which is similar to this study. Additionally, mobile phone ownership has been shown to play an important role in cholera interventions and prevention strategies^81^ and our results corroborate similar findings that a higher prevalence of mobile phone ownership results in reduced cholera risk as it enhances communication and information dissemination, which can lead to better hygiene practices and faster response to cholera outbreaks. An example of this was shown in Mozambique where a short message service was used to gather information to support cholera response activities and vaccination campaigns.^81^

We have shown that temperature precipitation, NDVI and RH contributed to increased cholera risk at different lag periods in Mozambique. The results of the study highlight the need for identifying vulnerable populations to further support cholera control efforts and the utility of combining climate, environmental conditions, regional spatial stratification, socioeconomic factors and public health interventions related to cholera risk. Appropriate interventions for control and elimination of cholera in Mozambique require a multidisciplinary innovative approach, to prevention and sustained political commitment at national, provincial and district levels, as well as continued investment in improving the availability of WASH services and infrastructure with appropriate quality and quantity across urban and rural areas of the country.

Results from our current study support the uptake of measures that can also help achieve the Sustainable Development Goals, especially those related to (1) good health and well-being, (2) clean water and sanitation and (3) climate action.

This study had some limitations that need to be acknowledged. The cross-sectional nature of the Demographic and Health Survey (DHS) data limits our ability to draw precise conclusions about their influence on cholera transmission in time. Since DHS data are collected every few years, it may not accurately capture the temporal dynamics of cholera transmission and outbreak patterns within the study area. This temporal mismatch means that the data often do not coincide with the actual periods of cholera occurrence.

Spatial variability is another limitation, as the cholera, climate and DHS data were aggregated to the broader provincial level in our study. This aggregation may have masked the important local-level variations in cholera risk and transmission dynamics. Consequently, our analysis may have overlooked important microlevel heterogeneities that are critical for understanding the nuanced patterns of cholera spread and identifying targeted intervention strategies. Additionally, the study’s reliance on secondary sources of socioeconomic data such as the DHS limits the inclusion of certain key indicators such as infrastructural factors, for example, water quality, sanitation infrastructure and healthcare access.

Our study offers important contributions to understanding cholera transmission, the limitations related to the cross-sectional nature of the DHS data, potential recall bias, spatial aggregation, lack of certain key variables and inability to assess long-term trends must be carefully considered when interpreting the findings.

## Conclusions

This study explored lag patterns of monthly climate variables and cholera morbidity in Mozambique. Results show how the use of climate variables could help in the early warning system and control of cholera. The public health measures for the prevention and control of cholera occurrence must factor in climate considerations if it is to reduce vulnerability and increase the adaptive capacity of the population in Mozambique. Evidence presented here can support the planning, monitoring and evaluation of cholera control efforts.

## supplementary material

10.1136/bmjopen-2023-082503 online supplemental file 1

## Data Availability

Data are available on reasonable request.
