# Supplementary material for: Spatial-temporal analysis of climate and socioeconomic conditions on cholera incidence in Mozambique from 2000 to 2018: an ecological longitudinal retrospective study
Source: BMJ Open. 2024 Aug 19;14(8):e082503. doi: 10.1136/bmjopen-2023-082503 (PMC11337674; doi:10.1136/bmjopen-2023-082503)
Supplement: online supplemental file 1 [file bmjopen-14-8-s001.docx]

**Spatial temporal analysis of climate and socio-economic conditions on cholera incidence in Mozambique from 2000-2018: an ecological longitudinal retrospective study**

Chaibo Jose Armando**^1,8*^**, Joacim Rocklov **^1,2^**, Mohsin Sidat**^3^**, Yesim Tozan**^4^**, Alberto Francisco Mavume**^5^**, Aditi Bunker **^6,7^**, Maquins Odhiambo Sewe**^1,6^**

**^1^**Department of Public Health and Clinical Medicine, Umeå University, Section of sustainable health, Umeå, Sweden,

**^2^**Heidelberg Institute of Global Health & Interdisciplinary Centre for Scientific Computing, Heidelberg University, Heidelberg, Germany

**^3^**Faculty of Medicine, Eduardo Mondlane University, Maputo, Mozambique,

**^4^**School of Global Public Health, New York University, New York, NY, United States

**^5^**Faculty of Science, Eduardo Mondlane University, Maputo, Mozambique

**^6^** Heidelberg Institute of Global Health, University of Heidelberg, Heidelberg, Germany

**^7^** Center for Climate, Health, and the Global Environment, Harvard T.H. Chan School of Public Health, Boston, United States.

^8^Center for African Studies, Eduardo Mondlane University, Maputo, Mozambique.

* Corresponding author

E-mail: [cjarmando.jose@gmail.com](mailto:cjarmando.jose@gmail.com)

**Abstract**

**Objectives** To assess both socio-economic and climatic factors of cholera morbidity in Mozambique considering both spatial and temporal dimensions.

**Design** An ecological longitudinal retrospective study using monthly provincial cholera cases from Mozambican Ministry of Health between 2000-2018. The cholera cases were linked to social-economic data from Mozambique Demographic and Health Surveys conducted in the period 2000-2018 and climatic data; relative humidity (RH), mean temperature, precipitation, and Normalized Difference Vegetation Index (NDVI). A negative binomial regression model in a Bayesian framework was used to model cholera incidence while adjusting for the spatio-temporal covariance, lagged effect of environmental factors and the socio-economic indicators.

**Setting** Eleven provinces in Mozambique.

**Results** Over the 19-years period a total of 153,941 cholera cases were notified to the surveillance system in Mozambique. Risk of cholera increased with higher monthly mean temperatures above 24^o^C in comparison to the reference mean temperature of 23^o^C. At mean temperature of 19°C, cholera risk was higher at a lag of 5-6 months. At a shorter lag of 1 month, precipitation of 223.3 mm resulted in an 57% increase in cholera risk (RR 1.57 [95% CI: 1.06 - 2.31]). Cholera risk was greatest at 3 lag months with monthly NDVI of 0.137, (RR 1.220 [95% CI: 1.042 - 1.430]), compared to the reference value of 0.2. At an RH of 54%, cholera relative risk was increased by 62%, (RR 1.620 [95% CI: 1.124 - 2.342]) at a lag of 4 month. We found that ownership of radio RR 0.29, (95% CI: 0.109 - 0.776) and mobile phones RR 0.262 (95% CI: 0.097 - 0.711) were significantly associated with low cholera risk.

**Conclusion** The derived lagged patterns can provide appropriate lead times in a climate driven cholera early warning system that could contribute for the prevention and management of outbreaks.

**STRENGTHS AND LIMITATIONS OF THIS STUDY**

- Employs an ecological longitudinal retrospective design to analyze temporal and spatial trends in cholera incidence, yielding insights into the dynamics.
- The method comprehensively elucidates cholera incidence in Mozambique by exploring the influence of climate and socio-economic conditions employing advanced statistical techniques to explore the complex relationships.
- Understanding lagged effects of climate and socio-economic factors is essential for discerning the temporal delay between risk exposure and cholera incidence, offering insights into long-term dynamics for effective management.
- The use of cross-sectional DHS data, collected every five years and aggregation to provincial may have masked the relationship with cholera

**Introduction**

Cholera is a water-borne disease caused by the bacteria vibrio cholerae with the whole population at risk. Humans-infection of cholera mostly occur through faecal oral route through the ingestion of contaminated food or water [1-3]. Improved access to water, sanitation, and hygiene (WASH) has been shown to decrease cholera occurrence by interrupting transmission routes [4-7]. However, cholera continues to affect vulnerable populations in low resource settings with inadequate water and sanitation infrastructure. [8-10]. Cholera imposes a substantial global burden, with an annual estimated incidence of 1.3 to 4 million cases worldwide and results in high mortality rates, ranging from 21,000 to 143,000 deaths each year [4, 11-13]. Globally, the number of cholera cases showed a significant decline from 2014 to 2017. In 2015, there was a 9.49% reduction in reported cases compared to the 190,549 cases in 2014. This downward trend continued with a further 30.66% decrease in 2016, followed by an additional 37.02% decline in 2017. These figures indicate a consistent and substantial reduction in cholera cases worldwide over this period [11, 14]. Cholera remains endemic in sub-Saharan Africa (SSA) [15] and more than 87 million people live in areas with high risk of cholera transmission [16]. Between 1970-2011, there were a total of 3,221,050 cholera cases, and 9.79% (315,295) of the cases were reported in Mozambique [17]. Between 2000-2015, 83% of the cholera mortalities reported by the WHO occurred in Sub-Saharan Africa [18]. In 2017, there were 3,220 deaths among the reported 179,835 cases ,a case fatality rate (CFR) of 1.8% [19]. Sub-Sahara Africa including Mozambique experience year-around transmission of cholera [19, 20]. Mozambique registered the first cholera cases in early 1973 [21, 22]. Notified cholera cases from Mozambique account for about one fifth to one third of all cases reported in Africa across the years [21, 23]. In 2019, Sofala Province in Mozambique reported 6,766 cholera cases with 8 associated fatalities [24]. According to the United Nations Office for the Coordination of Humanitarian Affairs (OCHA) report, Mozambique recorded approximately 26,841 cholera cases and 123 fatalities in April 2023 [25].

The ongoing cholera burden in Mozambique is attributed to inadequate sanitation and extreme weather events, such as tropical cyclones and droughts [26-29] though the proportion of the population who have access to adequate sanitation in Mozambique has increased from 28% in 2015 [27] to 38% in 2022 [30]. Studies have investigated how WASH improvements can reduce the risk of cholera transmission in Mozambique [31, 32], Congo [33], Niger [34] and Kenya [35] and yet the effectiveness of these interventions has been not established [36]. The key association between cholera risk and WASH factors can help to identify and prioritize areas with the highest need for intervention [37-39]. In addition to the WASH factors, cholera transmission is affected by various climatic factors such as temperature and precipitation [40-42]. Temperature is considered as an important factor for vibrio cholerae occurrence and propagation in the water reserves such as river, lake, oceans, stagnated water, and ground water [43, 44]. Furthermore, precipitation influence cholera transmission by increasing the risk of water contamination and concentration of vibrio cholerae through runoff [45, 46]. These climate factors affect the temporal and spatial range of the *Vibrio cholerae* and influence exposure pathways [47]. In Mozambique, cholera transmission generally peaks between December and April during the hot and rainy season [48, 49].

Mozambique is a low-income country with weak WASH infrastructure and limited resources to scale up interventions against cholera outbreaks. Cholera has been linked to the households with low socioeconomic conditions, poverty, inequalities, education level and poor sanitation [31, 50]. The incubation period of cholera is relatively short ranging from just 2 hours to 5 days, thus the cases can increase quite substantially in a relatively short time [51, 52]. Identification of vulnerable areas for multisectoral interventions can be a great first step for cholera control and mitigation [53]. Several interventions are used for cholera control and prevention including distribution of oral cholera vaccine, improved drinking water and sanitation facilities for communities at risk areas, as well as provisions of adequate health care [54, 55]. In this study, we aim to assess how climate and socio-economic conditions affect cholera incidence in Mozambique considering the lagged patterns over space and time. Through analysis of temporal lag patterns, we can better understand and respond to the dynamics of cholera transmission, leading to more effective prevention and control strategies.

**Materials and Methods**

**Study area**

Mozambique is one of the most vulnerable African countries to climate change along its coasts [56], with the majority of people living in rural areas along the coast [57]. Mozambique is bounded by Tanzania on the north, Malawi, and Zambia on the north-west, Zimbabwe on the west, South Africa and Swaziland on the south-west and the Indian Ocean on the east (see Supplemental S1 Figure).

**Data**

Weekly cholera cases at province level were extracted from the Mozambique Ministry of Health disease surveillance system for the years 2000-2018, and aggregated to monthly totals. Data on the climatic factors, including daily precipitation, relative humidity (RH), normalized different vegetation index (NDVI), and minimum and maximum temperature ($T_{min}$, $T_{max}$), were sourced from National Center for Environmental Prediction (NCEP) [58].

The NCEP employs advanced computer models that integrate observational data to simulate and predict weather patterns, including temperature, precipitation, NDVI and RH. These diverse data collection methods enable NCEP to generate reliable and detailed climate data which is available globally at different resolutions [59]. Monthly summaries of the daily climatic data were computed and summarised at provincial level based on shapefiles [60]. In addition, the population data used in the analysis was sourced from Woldpop [61].

Socioeconomic data from the Demographic Health Surveys (DHS) for the years 2003, 2009 and 2015 were included in the analysis [62]. The socio-economic variables included in this study followed similar variables used in [63] and were aggregated to the provincial level applying the method used in [63], (see Supplemental S1 and S2 Tables).

**Patient and public involvement**

No patients or public were involved in the design, conduct, reporting, or dissemination for this study.

**Statistical analysis**

We employed an ecological longitudinal retrospective study design to analyze the temporal and spatial pattern of cholera incidence rates in Mozambique. We applied Distributed lag non-linear models (DLNM) [64] in a Bayesian framework with Integrated Nested Laplace Approximation (INLA) [65] to model the delayed and non-linear relationship between minimum temperature ($T_{min}$), mean temperature ($T_{mean}$), maximum temperature ($T_{\max}$), total precipitation, RH, and NDVI and cholera incidence adjusting for the DHS derived socio-economic indicators. In DLNM methodology, a bi-dimensional crossbasis function is created in order to simultaneously capture the lag and the variable dimension spaces [64]. Natural cubic spline with three degrees of freedom was used for both the lag and the variable dimension. Lags of 0-6 months for climatic variables were assessed. The over-dispersed cholera cases were modelled using a negative binomial distribution. Backward elimination based on significance at 95% credible interval was used to select the socio-economic variables in the final model.

A significance level of 5% means that there is a 5% chance of incorrectly rejecting the null hypothesis. The final model consisted of the crossbasis functions of the climatic variables and the significant socioeconomic factors.

We used the following reference values 18^o^C, 23^o^C, 28^o^C, 77 mm, 73%, 0.2 for $T_{min}$, $T_{mean}$, $T_{max}$, precipitation, RH and NDVI respectively when defining crossbasis functions. Interpretations are made in reference to these values.

The final model selected is represented in equation (1) below:

$$Y_{it}\sim Negbin$$

$$log(Y_{it)}=\beta_{o}+\left( u_{i}+\nu_{i} \right)+\omega_{t}+f\left( x_{j},vardf,lagdf \right)+log(E_{t})\left( 1 \right)$$

$Y_{it}$ represents cholera incidence rate for province $i$ in month $t$, $\beta_{o}$ is the intercept,$u_{i}$ is the provincial exchangeable area effect while $\nu_{i}$ is the spatially structured random area effect.$\omega_{t}$ is the monthly random effect modeled with random walk of order 1. $f\left( x_{j},vardf,lagdf \right)$ represents the defined crossbasis function for climate and socio-economic variable $x_{j}$ with vardf and lagdf representing the degrees of freedoms for the variable and lag basis function. $E_{t}$ is the population offset added to the model with a coefficient of 1.

We used default INLA prior specifications for the provincial and monthly random effects.

All the analysis were done using R version 4.2.0 [66] while DLNM [64] and INLA [65] packages in R were used for the analysis.

**Results**

**Cholera cases and environmental variables**

There were 153,941 cholera cases reported between 2000 and 2018 in the whole of Mozambique. The annual cholera incidence rates ranged from a high of 181.5 in year 2002 to a low of 1.8 cases per 100,000 population in year 2014 (see Supplemental S3 and S4 Tables). In the periods 2005-2009, 2010-2014, and 2015-2018, Mozambique experienced huge reductions in cholera incidence rates. For example, between 2005 and 2009, cholera incidence rate decreased by 68.91% from 531.2 in the baseline period 2000-2004 to 167.3 per 100,000 population. This downward trend continued in 2010-2014, with a further reduction of 92.54% to a rate of 40.1 per 100,000. By 2015-2018, cholera incidence rate had decreased by 92.74% compared to the 2000-2004 period to 39.1 per 100000 (Table 1).

**Table 1** Summary of monthly cholera cases and environmental factors in Mozambique from 2000-2018.

|  | **2000-2018** | **2000-2004** | **2005-2009** | **2010-2014** | **2015-2018** |
| --- | --- | --- | --- | --- | --- |
| **Cholera Cases** | | | | | |
| Min | 0 | 11 | 0 | 0 | 0 |
| Max | 8349 | 8349 | 5579 | 1529 | 1750 |
| Mean (SD) | 675.18(1,408.68) | 1,612.95(2203.69) | 613.42(1099.62) | 160.68(305.78) | 223.29(422.78) |
| **Cholera Incidence Rate per 100,000** | | | | | |
| Min | 0 | 0.61 | 0 | 0 | 0 |
| Max | 494.70 | 494.70 | 270.73 | 72.17 | 71.85 |
| Mean (SD) | 37.00(81.12) | 95.38(130.24) | 30.67(53.65) | 7.37(14.17) | 8.96(17.26) |
| **Minimum Temperature** | | | | | |
| Min | 13.32 | 14.09 | 13.96 | 13.32 | 14.50 |
| Max | 23.0 | 22.75 | 23.0 | 22.69 | 22.76 |
| Mean (SD) | 18.98(2.94) | 19.14(2.92) | 19.11(3.00) | 18.70(3.03) | 18.97(2.83) |
| **Mean Temperature** | | | | | |
| Min | 18.65 | 18.98 | 19.20 | 18.65 | 19.59 |
| Max | 27.34 | 26.84 | 27.34 | 26.99 | 27.34 |
| Mean (SD) | 23.73(2.44) | 23.64(2.46) | 23.98(2.45) | 23.47(2.50) | 23.85(2.37) |
| **Maximum Temperature** | | | | | |
| Min | 23.65 | 23.66 | 24.36 | 23.65 | 24.59 |
| Max | 31.92 | 31.30 | 31.74 | 31.56 | 31.92 |
| Mean (SD) | 28.48(2.06) | 28.14(2.11) | 28.85(2.00) | 28.24(2.06) | 28.74(2.01) |
| **Normalized Different Vegetation Index** | | | | | |
| Min | 0.121 | 0.121 | 0.171 | 0.165 | 0.129 |
| Max | 0.365 | 0.365 | 0.343 | 0.349 | 0.331 |
| Mean (SD) | 0.240(0.050) | 0.240(0.050) | 0.250(0.060) | 0.240(0.050) | 0.230(0.050) |
| **Relative Humidity** | | | | | |
| Min | 59.17 | 60.41 | 59.79 | 59.38 | 59.17 |
| Max | 91.48 | 91.48 | 89.79 | 89.52 | 88.62 |
| Mean (SD) | 73.88(9.19) | 74.86(9.26) | 72.75(9.18) | 74.15(9.35) | 73.72(9.03) |
| **Precipitation** | | | | | |
| Min | 1.35 | 2.42 | 2.48 | 1.35 | 2.89 |
| Max | 345.01 | 345.01 | 262.25 | 344.84 | 260.31 |
| Mean (SD) | 77.60(80.85) | 90.28(90.52) | 74.68(79.34) | 73.79(78.48) | 70.18(73.20) |

The sea­sonal variation in cholera incidence rates followed the seasonal variation in environmental factors (Figure 1, see Supplemental S2 Figure). During the period 2000-2018, cholera incidence rate decreased, and cholera burden was geographically concentrated in the central and northern parts of Mozambique (Figure 1, see Supplemental S3-C and S4 Figures). The year 2014 had the lowest national average of 40 cholera cases per month while the year 2002 had the highest mean of about 2809 cases, as shown in Supplemental S4 Table. Cholera outbreaks in Mozambique show strong seasonal pattern with an increased burden from November to April (Figure 1). Cholera seasonality in Mozambique varies geographically, with peak transmission occurring earlier in the year in the northern regions, such as Cabo Delgado and Nampula provinces, and central regions, including Zambezia, Sofala, and Manica provinces (see Supplemental S4 Figure). Over the study period, the northern and central regions of Mozambique received more precipitation compared to other parts of the country. The driest provinces were Maputo, Gaza, and Maputo City (see Supplemental S5 Figure). The highest temperatures (minimum, mean, and maximum) are observed along the central and northern coast, while the lowest temperatures occur in Gaza and Maputo province (see Supplemental S6-8 Figures). Mozambique exhibits a simple seasonal temperature profile, with the lowest temperatures in July and the highest in December. Between 2000 and 2018, the annual mean relative humidity in Mozambique ranged from 63.6% to 79.8% (see Supplemental S9 Figure), indicating significant fluctuations in atmospheric moisture levels over the years. Lower NDVI values are seen in Maputo City, Gaza, Maputo, Tete, and Niassa provinces (see Supplemental S10 Figure), while the higher NDVI values are found in the central provinces such as Sofala, Manica, and Zambezia (see Supplemental S10 and S11 Figures).

Socio-economic indicators

Summaries of the DHS derived socio-economic and WASH factors aggregated at the provincial level are shown in Supplemental S1 Table.

Specifically considering access to clean water and adequate sanitation, two key promoters of overall health and wellbeing, majority of households (56.8%) in the provinces reported no access to treated drinking water (see Supplemental S1 Table). The proportion of households that shared a toilet facility with other households differed across the provinces ranging from 4-17% with an average of 10.1%. The average proportion of households with radio and mobile phone ownership was 39%,67% with a range of 27-55% and 45-97% respectively. As for educational attainment, on average, 25.1% of households in the provinces reported no education, while the highest was 38.6% (see Supplemental S1 Table).

Model results

Temperature

Table 2 displays the relationship between mean temperature and cholera risk at different lags and percentiles. The relative risk (RR) of cholera gradually increased as minimum, mean and maximum temperature increased (Figure 2A, see Supplemental S12 Figure). We observed lower risk of cholera for mean temperatures between 19-22°C at lags 0-4 months, compared to the reference of 23°C. The highest relative risk (RR) of cholera was at 28°C with a lag of 0 months (Table 2). At 19°C, cholera risk was also high compared to that the reference but with a much longer lag of 5-6 months. Lower risk of cholera were, however, observed at much shorter lags. We observed an increasing RR of cholera for mean temperature above 24°C (Table 2). We observed an increased risk of cholera at maximum temperatures between 24-33°C with lags of 2-3 months, compared to the reference value of 28°C, although this finding was not statistically significant (see Supplemental S5 Table).

**Table 2** The association between mean temperature, precipitation, relative humidity, NDVI and cholera risk at lags 0-6 months.

| **Lag** | **Mean temperature (Ref=23^o^C)** | | | | | | | |
| --- | --- | --- | --- | --- | --- | --- | --- | --- |
|  | **Percentile** | | | | | | | |
|  | **5^th^** | **10^th^** | **25^th^** | | **50^th^** | **75^th^** | **90^th^** | **99^th^** |
|  | **19** | **20** | **22** | | **24** | **26** | **27** | **28** |
| 0 | 0.72 | 0.70 | 0.85 | | 1.15 | 1.28 | 1.24 | 1.54 |
| 1 | 0.79 | 0.78 | 0.90 | | 1.11 | 1.22 | 1.21 | 1.19 |
| 2 | 0.87 | 0.87 | 0.93 | | 1.10 | 1.17 | 1.20 | 1.22 |
| 3 | 0.94 | 0.94 | 0.97 | | 1.04 | 1.14 | 1.19 | 1.25 |
| 4 | 1.00 | 1.00 | 0.99 | | 1.02 | 1.12 | 1.20 | 1.29 |
| 5 | 1.07 | 1.05 | 1.00 | | 1.01 | 1.11 | 1.21 | 1.33 |
| 6 | 1.13 | 1.10 | 1.02 | | 1.00 | 1.12 | 1.23 | 1.37 |
|  | **Precipitation (Ref=77 mm)** | | | | | | | |
|  | **1.2** | **2.4** | | **7.4** | **33.7** | **114.5** | **223.3** | **395.1** |
| 0 | 0.75 | 0.76 | | 0.77 | 0.85 | 1.14 | 1.57* | 1.80 |
| 1 | 0.78 | 0.78 | | 0.79 | 0.87 | 1.22* | 1.46* | 1.53 |
| 2 | 0.86 | 0.86 | | 0.87 | 0.92 | 1.07 | 1.21 | 1.12 |
| 3 | 0.99 | 0.99 | | 0.99 | 0.99 | 1.00 | 0.95 | 0.81 |
| 4 | 1.05 | 1.05 | | 1.05 | 1.03 | 0.97 | 0.88 | 0.77 |
| 5 | 0.96 | 0.96 | | 0.96 | 0.98 | 1.02 | 1.02 | 0.91 |
| 6 | 0.76 | 0.77 | | 0.78 | 0.86 | 1.12 | 1.37 | 1.15 |
|  | **Relative humidity (Ref=73%)** | | | | | | | |
|  | **54** | **61** | | **67** | **74** | **82** | **88** | **92** |
| 0 | 0.56 | 0.61 | | 0.78 | 1.03 | 1.13 | 1.00 | 0.89 |
| 1 | 0.85 | 0.87 | | 0.92 | 1.01 | 1.08 | 1.09 | 1.09 |
| 2 | 1.22 | 1.16 | | 1.07 | 0.99 | 1.04 | 1.18 | 1.32 |
| 3 | 1.53* | 1.40* | | 1.17 | 0.98 | 1.03 | 1.26 | 1.50 |
| 4 | 1.62* | 1.45* | | 1.19* | 0.98 | 1.04 | 1.31 | 1.62 |
| 5 | 1.51* | 1.36 | | 1.15 | 0.98 | 1.07 | 1.35 | 1.65 |
| 6 | 1.32 | 1.20 | | 1.08 | 0.99 | 1.11 | 1.37 | 1.64 |
|  | **NDVI (Ref=0.2)** | | | | | | | |
|  | **0.137** | **0.157** | | **0.189** | **0.238** | **0.295** | **0.337** | **0.400** |
| 0 | 1.02 | 1.00 | | 1.00 | 0.99 | 1.00 | 1.03 | 1.09 |
| 1 | 1.12 | 1.09 | | 1.03 | 0.91 | 0.81 | 0.79 | 0.83 |
| 2 | 1.20* | 1.15* | | 1.04* | 0.85* | 0.69 | 0.65* | 0.67 |
| 3 | 1.22* | 1.17* | | 1.05* | 0.83* | 0.65* | 0.59* | 0.59* |
| 4 | 1.16* | 1.13* | | 1.03* | 0.87* | 0.70* | 0.63* | 0.59* |
| 5 | 1.05 | 1.04 | | 1.01 | 0.94 | 0.84 | 0.76 | 0.65 |
| 6 | 0.93 | 0.94 | | 0.98 | 1.05 | 1.06 | 0.97 | 0.74 |

*Significant

**Precipitation**

Table 2 shows the estimated relationship between precipitation and relative cholera risk at lags 0-6 months. There were delayed effects on cholera risk with precipitation, with the maximum effects of heavy precipitation reached at shorter lags of less than 1 month. For example, total precipitation of 223.3 mm at with lag of 1 month, a resulted in 57% increase in cholera risk (RR 1.57 [95% CI: 1.06 - 2.31]). We also observed that the effect of heavy precipitation on cholera risk was attenuated at lags 3-5 months. The overall association between cumulative precipitation and cholera risk is displayed in Figure 2B. Increasing amounts of precipitation were associated with increased cholera risk at lags 0-2 months (Table 2). The RR of cholera decreased as precipitationincreased at lag 4 months (Table 2). At precipitation of above 500 mm, cholera risk was lower (Figure 2B).

**Relative humidity**

Table 2 shows exposure-lag response surface for RH and cholera risk. For RH between 54% and 67%, the risk of cholera increased at longer lags for RH of 54% (RR 1.620 [95% CI: 1.124 - 2.342]) at lag 4 month. This association is more intense at a lag of 3 to 5 months. Higher RH values was found to be significantly associated with cholera risk at longer lags. At an RH of 67%, cholera risk increased by 19%, (RR 1.190 [95% CI: 1.017 - 1.392] at a lag of 4 month. The non-linear relationship between RH and cholera risk is shown in Figure 2C. For RH greater than 80% and below 70%, cholera risk consistently increased though the association was not statistically significant (Figure 2C).

Normalized difference vegetation Index (NDVI)

Table 2 displays the lag-response association between NDVI and cholera risk. For NDVI of 0.137, cholera risk was highest at a lag of 3 months (RR 1.220 [95% CI: 1.042 - 1.430]). We observed a decreased relative risk for NDVI values from 0.238 to 0.400 compared to the reference value of 0.2, for example at NDVI value of 0.337 cholera risk was 37% lower (RR 0.630 [95% CI: 0.468 - 0.857]), compared to the reference. The overall relationship between NDVI and cholera risk is displayed in the Figure 2D. In comparison to the reference value of 0.2, cholera risk was significantly lower for NDVI value above 0.22 (Figure 2D). Specifically, at a monthly mean NDVI of 0.38, cholera risk was 88.9% lower, (RR 0.110 [95% CI: 0.012-0.984]), and at 0.27, it was 66.78% lower (RR 0.332 [95% CI: 0.118 - 0.928]) (Figure 2D). For NDVI values below the reference value of 0.2, the risk of cholera slightly increased though it did not show a significant relationship with cholera risk (Figure 2D).

**Socio-economic factors for cholera**

Table 3 shows the association between household socio-economic indicators and cholera risk. A high proportion of households with a radio (used for delivering outbreak messages) was significantly associated with very low risk ,70.1% lower, (RR 0.29 [95% CI: 0.109 - 0.776]), compared to having low percentage of radio ownership. Similarly, high proportion of mobile phones ownership was associated with 73.76% decreased risk of cholera compared households with low ownership, (RR 0.262 [95% CI: 0.097 - 0.711]). Conversely, we observed an increased risk of cholera in households sharing a toilet, though this was not statistically significant.

**Table 3** The association between socio-economic variables and cholera risk. The reference value from household with a radio, mobile phone and whose who shares toilet facility is **26.68%**, **45%** and **7%** respectively.

|  | **Radio ownership (Ref=26.68%)** | | | | | |
| --- | --- | --- | --- | --- | --- | --- |
|  | **Percentile** | | | | | |
|  | **5^th^** | **10^th^** | **25^th^** | **50^th^** | **75^th^** | **90^th^** |
|  | **27** | **37** | **39** | **40** | **41** | **50** |
| RR | 1.01 | 0.56 | 0.39 | 0.33* | 0.28* | 0.29* |
|  | **Mobile phone ownership (Ref=45%)** | | | | | |
|  | **45.4** | **45.7** | **46.9** | **69.9** | **86.1** | **87.9** |
| RR | 0.997 | 0.994 | 0.986 | 0.697 | 0.262* | 0.292* |
|  | **Toilet sharing (Ref=7%)** | | | | | |
|  | **4.02** | **5.02** | **7.76** | **9.23** | **13.13** | **14.2** |
| RR | 1.096 | 1.034 | 1.043 | 1.212 | 1.302 | 1.108 |

*Significant

**Discussion**

We analysed the non-linear relationship between delayed climatic conditions and cholera risk in Mozambique while adjusting for socio-economic conditions, space and time dependencies in a Bayesian framework making inference using the computationally efficient INLA methodology. We show that temperature, precipitation, relative humidity (RH) and NDVI influence the spatiotemporal distribution of cholera infections in Mozambique.

Meteorological factors play a crucial role in cholera transmission pathways, for example high temperatures in a warmer season may provide a suitable condition for Vibrio cholerae to proliferate [42-44, 67]. In this study, we found that mean temperature above 24 °C increased cholera risk at lags 0-6 months. At mean temperatures between 19°C and 22°C, the risk was lower at shorter lags. A study looking at countries in SSA linked elevated cholera risk with mean temperature at lags above 2 months [1] which is consistent with our study. In Zanzibar, cholera risk increased with 1^o^C rise in temperature with a delay of 4 months [68]. In Tanzania, they also found significant relationship between temperature and cholera risk, however they did not consider the lagged effect of temperature [69].

Similar to temperature, precipitation also modulates cholera risk acting at different spatial and temporal scales. In this study, we found that increased precipitation was associated with increased risk of cholera occurrence at a 1-month lag. With monthly precipitation of between 150-500 mm, the risk of cholera was high at shorter lag of 1 month and reduced at higher lags of 3 to 5 months. This resonates with our finding as the incubation period for Vibrio cholerae causing cholera, is usually less than a month with most symptoms appearing within 1-3 days after ingestion of contaminated food or water. In South Sudan, precipitation was found to be the most important driver for cholera ,however this study also did not explore the delayed effect of precipitation [45]. In Bangladesh, a significant one-month delay, similar to our finding was found between precipitation and cholera risk where a 1 mm/day increase in mean precipitation was linked with an 6.5% increase in cholera transmission [70], while in this study we found increase of 57% at precipitation of 223.3. In Zambia, higher cholera risk was found at a lag less than one months for precipitation above 50 mm [71]. Though in South Africa [72], precipitation had the strongest association with cholera risk at 2-month lag, while in Haiti [73] shorter lags less than 1-month were found , very similar our findings.

We found significant association between NDVI values and cholera risk, with lower NDVI values linked to higher cholera risk and higher NDVI values associated with reduced risk. Lower NDVI values are often associated with dry or drought conditions, which can exacerbate cholera transmission. During droughts, water scarcity forces populations to rely on contaminated water sources, such as stagnant ponds, rivers and lakes for drinking and household purposes, thereby increasing the risk of cholera transmission. NDVI is the most used vegetation index in different areas including epidemiology and climate variability [74]. NDVI increases with the amount of green biomass and precipitation. In this study we found NDVI to be positively associated with precipitation.

Our findings indicated that RH levels between 54% and 67% were linked to an increased risk of cholera, with effects observed after delays ranging from 0 to 6 months. Specifically, the risk of cholera was notably higher when longer lag periods were considered within this RH range.

This is consistent with the study done in India [75]. Similarly, in Zanzibar, cholera outbreaks were found to be significantly associated with RH at a lag of 5 months [68]. Relative humidity (RH) critically influences cholera transmission dynamics by modulating the survival of Vibrio cholerae bacteria in environmental reservoirs and impacting human susceptibility to infection. RH directly influences the persistence of Vibrio cholerae in various water sources, including rivers, ponds, and coastal areas, potentially extending the bacterium's survival duration in water and thereby augmenting the risk of exposure through contaminated water sources.

Household’s socioeconomic disparities can help elucidate the fluctuations in cholera transmission between and within provinces in Mozambique during the outbreak in addition to the climatic factors. Studies done in Zambia [76, 77] and Kenya [78, 79] showed that households sharing toilet facilities were at higher risk of cholera infections, a finding consistent with our study. Sharing toilet facilities increases cholera risk due to factors such as inadequate sanitation, increased cross-contamination, and limited access to hygiene resources. Shared toilet facilities often lack proper sanitation and maintenance, resulting in the accumulation of faecal matter and increased likelihood of faecal-oral transmission of Vibrio cholerae.

A study conducted in Haiti found that household with radio had lower cholera risk [80] which is similar with this study. Additionally, mobile phone ownership has been shown to play an important role in cholera interventions and prevention strategies [81] and our results corroborate similar findings that higher prevalence of mobile phone ownership results in reduced cholera risk as it enhances communication and information dissemination, which can lead to better hygiene practices and faster response to cholera outbreaks. Example this was shown in Mozambique where, short message service (SMS) was used to gather information to support cholera response activities and vaccination campaigns [81].

We have shown that temperature$,$precipitation, NDVI, and RH contributed to increased cholera risk at different lag periods in Mozambique. The results of the study highlight the need for identifying vulnerable populations to further support cholera control efforts and utility of combining climate, environmental conditions, regional spatial stratification, socioeconomic factors, and public health interventions related to cholera risk. Appropriate interventions for control and elimination of cholera in Mozambique requires a multidisciplinary innovative approach, in prevention, and sustained political commitment at national, provincial and district levels, as well as continued investment in improving the availability of water, sanitation, and hygiene (WASH) services and infrastructure with appropriate quality and quantity across urban and rural areas of the country.

Results from our current study support the uptake of measures that can also help achieve the Sustainable Development Goals (SDGs) especial related to (i) good health and well-being, (ii) clean water and sanitation, (iii) climate action.

This study had some limitations that need to be acknowledged. The cross-sectional nature of the Demographic and Health Survey (DHS) data limits our ability to draw precise conclusions about their influence on cholera transmission in time. Since DHS data is collected after every few years, it may not accurately capture the temporal dynamics of cholera transmission and outbreak patterns within the study area. This temporal mismatch means that the data often does not coincide with the actual periods of cholera occurrence.

Spatial variability is another limitation, as the cholera, climate and DHS data was aggregated to broader provincial level in our study. This aggregation may have masked the important local-level variations in cholera risk and transmission dynamics. Consequently, our analysis may have overlooked important micro-level heterogeneities that are critical for understanding the nuanced patterns of cholera spread and identifying targeted intervention strategies. Additionally, the study's reliance on secondary sources of socio-economic data such as the DHS limits inclusion of certain key indicators such as infrastructural factors e.g water quality, sanitation infrastructure, and healthcare access.

Our study offers important contributions to understanding cholera transmission, the limitations related to the cross-sectional nature of the DHS data, potential recall bias, spatial aggregation, lack of certain key variables, and inability to assess long-term trends must be carefully considered when interpreting the findings.

**Conclusions**

This study explored lag patterns of monthly climate variables and cholera morbidity in Mozambique. Results shows how use of climate variables could help in the early warning system and control of cholera. The public health measures for the prevention and control of cholera occurrence must factor in climate considerations if it is to reduce vulnerability and increase the adaptive capacity of the population in Mozambique. Evidence presented here can support the planning, monitoring, and evaluation of cholera control efforts.

**Contributions**

Conceived and designed the study: CA, MOS, JR. Analyzed the data: CA, JR, MOS. Methodology: CA, JR, MOS. Software: CA, MOS. Visualization: CA, MOS. Wrote the paper: CA, JR, MS, YT, AM, AB, MOS. Writing - review & editing: CA, JR, MS, YT, AM, AB, MOS. CA is responsible for the overall content as the guarantor.

**Acknowledgements**

The first author is thankful to the Ministry of Health in Mozambique for providing the data. We are deeply grateful to all those who played a role in the success of this research paper.

**Funding**

The study is funded by Swedish International Development Agency (SIDA). Not applicable for award/grant number.

**Competing interests**

The authors have declared that no competing interests exist.

**Patient and public involvement**

Not applicable.

**Patient consent for publication**

Not applicable.

**Ethics approval**

Not applicable.

**Provenance and peer review**

Not commissioned, externally peer reviewed.

**Data Availability**

The cholera datasets analyzed during the current study are available from the

corresponding author on reasonable request.

**Reference**

1. Perez-Saez, J., et al., *The seasonality of cholera in sub-Saharan Africa: a statistical modelling study.* Lancet Glob Health, 2022. **10**(6): p. e831-e839.

2. Cliff, J., P. Zinkin, and A. Martelli, *A hospital outbreak of cholera in Maputo, Mozambique.* Transactions of the Royal Society of Tropical Medicine and Hygiene, 1986. **80**(3): p. 473-476.

3. Goh, K., et al., *Person-to-person transmission of cholera in a psychiatric hospital.* Journal of Infection, 1990. **20**(3): p. 193-200.

4. Jones, N., et al., *Water, sanitation and hygiene risk factors for the transmission of cholera in a changing climate: using a systematic review to develop a causal process diagram.* Journal of Water and Health, 2020. **18**(2): p. 145-158.

5. Mukandavire, Z., et al., *Estimating the reproductive numbers for the 2008–2009 cholera outbreaks in Zimbabwe.* Proceedings of the National Academy of Sciences, 2011. **108**(21): p. 8767-8772.

6. YATES, T., et al., *Water, sanitation, and hygiene interventions in outbreak response: a synthesis of evidence.* Waterlines, 2018: p. 5-30.

7. Clemens, J., et al., *Cholera. The Lancet.* Cholera. The Lancet, 2017. **390**(10101).

8. Musoke, D., et al., *The role of Environmental Health in preventing antimicrobial resistance in low- and middle-income countries.* Environ Health Prev Med, 2021. **26**(1): p. 100.

9. Bwire, G., et al., *Epidemiology of cholera outbreaks and socio-economic characteristics of the communities in the fishing villages of Uganda: 2011-2015.* PLoS neglected tropical diseases, 2017. **11**(3): p. e0005407.

10. Sodjinou, V.D., et al., *The 2021 cholera outbreak in West Africa: epidemiology and public health implications.* Archives of Clinical and Biomedical Research, 2022. **6**(2): p. 296-307.

11. Organization, W.H., *World health statistics 2018: monitoring health for the SDGs, sustainable development goals*. 2018: World Health Organization.

12. Ali, M., et al., *Updated global burden of cholera in endemic countries.* PLoS neglected tropical diseases, 2015. **9**(6): p. e0003832.

13. Ali, M., et al., *The global burden of cholera.* Bulletin of the World Health Organization, 2012. **90**(3): p. 209-218.

14. mondiale de la Santé, O. and W.H. Organization, *Weekly Epidemiological Record, 2016, vol. 91, 38 [full issue].* Weekly Epidemiological Record= Relevé épidémiologique hebdomadaire, 2016. **91**(38): p. 432-440.

15. Zerbo, A., R.C. Delgado, and P.A. González, *A review of the risk of cholera outbreaks and urbanization in sub-Saharan Africa.* Journal of Biosafety and Biosecurity, 2020. **2**(2): p. 71-76.

16. Lessler, J., et al., *Mapping the burden of cholera in sub-Saharan Africa and implications for control: an analysis of data across geographical scales.* The Lancet, 2018. **391**(10133): p. 1908-1915.

17. Mengel, M.A., et al., *Cholera outbreaks in Africa.* Cholera outbreaks, 2014: p. 117-144.

18. WHO, *Number of reported deaths - Data by country. WHO.* 2023.

19. Organization, W.H., *Cholera, 2017 Weekly epidemiological record*. 2017: Geveva.

20. Legros, D., *Global cholera epidemiology: opportunities to reduce the burden of cholera by 2030.* The Journal of infectious diseases, 2018. **218**(suppl_3): p. S137-S140.

21. ORGANIZATION, W.R.H., *Global Task Force on Cholera Control CHOLERA COUNTRY PROFILE: MOZAMBIQUE. 15 July 2013*. *2013*: WHO.

22. WHO, *Cholera Country Profile: Mozambique. World Health Organization. Global Task on Cholera Control.* 2009.

23. Langa, J.P., et al., *Epidemic waves of cholera in the last two decades in Mozambique.* The Journal of Infection in Developing Countries, 2015. **9**(06): p. 635-641.

24. Mongo, E., E. Cambaza, and R. Nhambire, *Outbreak of cholera due to cyclone Idai in central mozambique (2019)*. 2019, IntechOpen.

25. OCHA, *Mozambique: Tropical Cyclone Freddy, Floods and Cholera Situation Report No. 2.* 2023. **2**.

26. Lequechane, J.D., et al., *Mozambique’s response to cyclone Idai: how collaboration and surveillance with water, sanitation and hygiene (WASH) interventions were used to control a cholera epidemic.* Infectious Diseases of Poverty, 2020. **9**(1): p. 1-4.

27. Borja-Vega, C., et al., *Findings of the Mozambique water supply, sanitation, and hygiene poverty diagnostic*. 2018, The World Bank.

28. OCHA, *Mozambique Response Plan: Cyclone Freddy, Floods & Cholera (March - September 2023).* 2023a.

29. Bié, A.J., et al., *Numerical modeling of storm surges in the coast of Mozambique: the cases of tropical cyclones Bonita (1996) and Lisette (1997).* Ocean Dynamics, 2017. **67**(11): p. 1443-1459.

30. UNICEF, and, and WHO, *Progress on household drinking water, sanitation and hygiene 2000–2022: special focus on gender*. 2023.

31. Collins, A., et al., *Socio‐economic and environmental origins of cholera epidemics in Mozambique: guidelines for tackling uncertainty in infectious disease prevention and control.* International journal of environmental studies, 2006. **63**(5): p. 537-549.

32. WHO, *Cholera - Mozambique.* 2023.

33. Gallandat, K., et al., *The impact of improved water supply on cholera and diarrhoeal diseases in Uvira, Democratic Republic of the Congo: a protocol for a pragmatic stepped-wedge cluster randomised trial and economic evaluation.* Trials, 2021. **22**(1): p. 1-17.

34. Graveleau, J., et al., *Influence of community-led total sanitation and water coverages in the control of cholera in Madarounfa, Niger (2018).* Frontiers in Public Health, 2021. **9**: p. 643079.

35. Kiama, C., et al., *Mapping of cholera hotspots in Kenya using epidemiologic and water, sanitation, and hygiene (WASH) indicators as part of Kenya’s new 2022–2030 cholera elimination plan.* PLOS Neglected Tropical Diseases, 2023. **17**(3): p. e0011166.

36. Taylor, D.L., et al., *The impact of water, sanitation and hygiene interventions to control cholera: a systematic review.* PLoS one, 2015. **10**(8): p. e0135676.

37. Ratnayake, R., et al., *Highly targeted spatiotemporal interventions against cholera epidemics, 2000–19: a scoping review.* The Lancet Infectious Diseases, 2021. **21**(3): p. e37-e48.

38. Burrowes, V., et al., *Risk factors for household transmission of vibrio cholerae in Dhaka, Bangladesh (CHoBI7 Trial).* The American Journal of Tropical Medicine and Hygiene, 2017. **96**(6): p. 1382.

39. Wolfe, M., et al., *A systematic review and meta-analysis of the association between water, sanitation, and hygiene exposures and cholera in case–control studies.* The American journal of tropical medicine and hygiene, 2018. **99**(2): p. 534.

40. Change, I.P.O.C., *Ipcc.* Climate change, 2014.

41. Islam, M.S., et al., *Environmental reservoirs of Vibrio cholerae.* Vaccine, 2020. **38**: p. A52-A62.

42. Usmani, M., et al., *Combating cholera by building predictive capabilities for pathogenic Vibrio cholerae in Yemen.* Scientific Reports, 2023. **13**(1): p. 2255.

43. Kruger, S.E., P.A. Lorah, and K.W. Okamoto, *Mapping climate change’s impact on cholera infection risk in Bangladesh.* PLOS Global Public Health, 2022. **2**(10): p. e0000711.

44. Asadgol, Z., et al., *The effect of climate change on cholera disease: The road ahead using artificial neural network.* PLoS One, 2019. **14**(11): p. e0224813.

45. Lemaitre, J., et al., *Rainfall as a driver of epidemic cholera: comparative model assessments of the effect of intra-seasonal precipitation events.* Acta tropica, 2019. **190**: p. 235-243.

46. Christaki, E., et al., *The impact of climate change on Cholera: A review on the global status and future challenges.* Atmosphere, 2020. **11**(5): p. 449.

47. UNICEF, *Cholera: A global call to action*. 2023.

48. Baltazar, C.S., et al., *Conditions to eliminate cholera in Mozambique-the pathway for the development of the national cholera plan.* The Pan African Medical Journal, 2022. **42**(279).

49. WHO, *Cholera in the WHO African Region. Weekly Regional Cholera Bulletin*. 2023, WHO: WHO - World Health Organisation

50. Das, R., et al., *Vibrio cholerae in rural and urban Bangladesh, findings from hospital-based surveillance, 2000–2021.* Scientific Reports, 2023. **13**(1): p. 6411.

51. Organization, W.H., *Ending cholera a global roadmap to 2030*, in *Ending cholera a global roadmap to 2030*. 2017. p. 32-32.

52. Azman, A.S., et al., *The incubation period of cholera: a systematic review.* Journal of Infection, 2013. **66**(5): p. 432-438.

53. Organization, W.H., *Cholera vaccines: WHO position paper.* Weekly Epidemiological Record= Relevé épidémiologique hebdomadaire, 2010. **85**(13): p. 117-128.

54. Zuckerman, J.N., L. Rombo, and A. Fisch, *The true burden and risk of cholera: implications for prevention and control.* The Lancet infectious diseases, 2007. **7**(8): p. 521-530.

55. Anetor, G. and F. Abraham, *Knowledge of cholera and its prevention amongst urban residents of a district in Abuja: The pivotal role of health education.* Research Journal of Health Sciences, 2020. **8**(2): p. 102-112.

56. INGC, *Main report: INGC Climate Change. Study on the Impact of Climate Change on Disaster Risk in Mozambique: Synthesis Report*, in  N.I.f.D. Management, Editor. 2009, INGC, Mozambique: Maputo-Mozambique.

57. UN-HABITAT, *Mozambique country brief 2023: To promote Sustainable urbanization in Mozambique as driver of socioeconomic Development, climate resilience and peace.* 2023.

58. NCEP. *NCEP-reanalysis II*. 2022 [cited 2022; Available from: <https://psl.noaa.gov/data/gridded/help.html#FTP>.

59. Campos, R.M. and R.M.d.J. Palmeira, *Analysis of Environmental Prediction*

*Data of Temperature and Relative Humidity*

*from NCEP/NOAA.* Int J Environ Sci Nat Res 27(4): IJESNR.MS.ID.556217 2021. **27**.

60. ROSEA, O., *MOZAMBIQUE ADMINISTRATIVE BOUNDARY COMMON OPERATIONAL DATABASE (COD-AB). .* OCHA, 2019.

61. Tatem, A.J., *WorldPop, open data for spatial demography.* Scientific data, 2017. **4**(1): p. 1-4.

62. DHS. *Survey search*. 2015; Available from: <http://dhsprogram.com/What-We-Do/survey-search.cfm> .

63. Armando, C.J., et al., *Climate variability, socio-economic conditions and vulnerability to malaria infections in Mozambique 2016–2018: a spatial temporal analysis.* Frontiers in Public Health, 2023. **11**: p. 1162535.

64. Gasparrini, A., B. Armstrong, and M.G. Kenward, *Distributed lag non‐linear models.* Statistics in medicine, 2010. **29**(21): p. 2224-2234.

65. Rue, H., S. Martino, and N. Chopin, *Approximate Bayesian inference for latent Gaussian models by using integrated nested Laplace approximations.* Journal of the Royal Statistical Society Series B: Statistical Methodology, 2009. **71**(2): p. 319-392.

66. R Core Team, A. and R.C. Team, *R: A language and environment for statistical computing. R Foundation for Statistical Computing, Vienna, Austria. 2012*. 2022.

67. Shackleton, D., et al., *Mechanisms of cholera transmission via environment in India and Bangladesh: state of the science review.* Reviews on Environmental Health, 2023(0).

68. Reyburn, R., et al., *Climate variability and the outbreaks of cholera in Zanzibar, East Africa: a time series analysis.* Am J Trop Med Hyg, 2011. **84**(6): p. 862-9.

69. Traerup, S.L., R.A. Ortiz, and A. Markandya, *The costs of climate change: a study of cholera in Tanzania.* Int J Environ Res Public Health, 2011. **8**(12): p. 4386-405.

70. Constantin de Magny, G., et al., *Environmental signatures associated with cholera epidemics.* Proc Natl Acad Sci U S A, 2008. **105**(46): p. 17676-81.

71. Luque Fernández, M.Á., et al., *Influence of temperature and rainfall on the evolution of cholera epidemics in Lusaka, Zambia, 2003–2006: analysis of a time series.* Transactions of the Royal Society of Tropical Medicine and Hygiene, 2009. **103**(2): p. 137-143.

72. Mendelsohn, J. and T. Dawson, *Climate and cholera in KwaZulu-Natal, South Africa: the role of environmental factors and implications for epidemic preparedness.* Int J Hyg Environ Health, 2008. **211**(1-2): p. 156-62.

73. Eisenberg, M.C., et al., *Examining rainfall and cholera dynamics in Haiti using statistical and dynamic modeling approaches.* Epidemics, 2013. **5**(4): p. 197-207.

74. Garai, S., et al., *Assessing correlation between Rainfall, normalized difference Vegetation Index (NDVI) and land surface temperature (LST) in Eastern India.* Safety in Extreme Environments, 2022. **4**(2): p. 119-127.

75. Rajendran, K., et al., *Influence of relative humidity in Vibrio cholerae infection: a time series model.* The Indian journal of medical research, 2011. **133**(2): p. 138.

76. DuBois, A., et al., *Epidemic cholera in urban Zambia: hand soap and dried fish as protective factors.* Epidemiology & Infection, 2006. **134**(6): p. 1226-1230.

77. Sasaki, S., et al., *Spatial analysis of risk factor of cholera outbreak for 2003–2004 in a peri-urban area of Lusaka, Zambia.* The American journal of tropical medicine and hygiene, 2008. **79**(3): p. 414-421.

78. Shultz, A., et al., *Cholera outbreak in Kenyan refugee camp: risk factors for illness and importance of sanitation.* The American journal of tropical medicine and hygiene, 2009. **80**(4): p. 640-645.

79. Mahamud, A.S., et al., *Epidemic cholera in Kakuma Refugee Camp, Kenya, 2009: the importance of sanitation and soap.* The Journal of Infection in Developing Countries, 2012. **6**(03): p. 234-241.

80. Grandesso, F., et al., *Risk factors for cholera transmission in Haiti during inter-peak periods: insights to improve current control strategies from two case-control studies.* Epidemiology & Infection, 2014. **142**(8): p. 1625-1635.

81. de Almeida, S., et al., *Enhancing Community Engagement Through Data Collection: Controlling the Cholera Epidemic in Mozambique.* 2020.

Figure Legend:

**Figure 1** Seasonal variation in the monthly cholera incidence rates (red) and monthly precipitation (light blue), minimum temperature (orange), mean temperature (black), maximum temperature (dark red), relative humidity (light green) and NDVI (green) in Mozambique from 2000-2018.

**Figure 2** Overall effect of (A), precipitation (B), relative humidity (C) and NDVI (D) on cholera risk in Mozambique, 2000-2018. The reference values for, precipitation, relative humidity and NDVI were 23° C, 77 mm, 73% and 0.2 respectively.
