# Supplementary material for: Spatial-temporal analysis of climate and socioeconomic conditions on cholera incidence in Mozambique from 2000 to 2018: an ecological longitudinal retrospective study
Source: BMJ Open. 2024 Aug 19;14(8):e082503. doi: 10.1136/bmjopen-2023-082503 (PMC11337674; doi:10.1136/bmjopen-2023-082503)
Supplement: Supplementary file 2 [file bmjopen-2023-082503.reviewer_comments.pdf]

PEER REVIEW HISTORY

BMJ Open publishes all reviews undertaken for accepted manuscripts. Reviewers are asked to complete a checklist review form and are provided with free text boxes to elaborate on their assessment. These free text comments are reproduced below.

ARTICLE DETAILS

**TITLE (PROVISIONAL)**	Spatial temporal analysis of climate and socio-economic conditions on cholera incidence in Mozambique from 2000-2018: an ecological longitudinal retrospective study
**AUTHORS**	Armando, Chaibo Jose; Rocklov, Joacim; Sidat, Mohsin; tozan, yesim; Mavume, Alberto Francisco; Bunker, A.; Sewe, Maquins Odhiambo

VERSION 1 - REVIEW

**REVIEWER NAME**	Luna, Expedito
**REVIEWER AFFILIATION**	University of Sao Paulo, Instituto de Medicina Tropical
**REVIEWER CONFLICT OF INTEREST**	None
**DATE REVIEW RETURNED**	14-Feb-2024

**GENERAL COMMENTS**	The authors report the results of an ecologic study (study design not mentioned) about the effects of climatic and social-economic variables on cholera incidence in Mozambique, in a 19-year period. They observed that temperature, precipitation, relative humidity, a vegatation index, sharing toilet facilities and owning a radio, were associated with the cholera risk.<br>The statistical methods were properly described and conducted. The discussion brings results from similar studies, with an updated bibliography.<br>I would suggest that the authors expand the discussion of the study's limitations. My particular concern is the issue of the ecological fallacy. By working with mean values, one would not be obscuring the existence of population groups at greater risk, or exposed to different climatic conditions? An additional concern is related to the effect of the social-econiomic variables on the cholera risk. Why "owning a radio" is associated with a lower risk, and a mobile phone with its increase?<br>My final question is related to the cholera time trend in Mozambique. How does the model explain the observed reduction of the incidence over time?

**REVIEWER NAME**	Moise, Kenny
**REVIEWER AFFILIATION**	Universite Quisqueya, Laboratoire Santé-Environnement
**REVIEWER CONFLICT OF INTEREST**	No competing interests
**DATE REVIEW RETURNED**	02-Apr-2024

**GENERAL COMMENTS**	The authors proposed a study to assess how climatic variability and socioeconomic conditions impact cholera incidence in Mozambique, while considering the lagged patterns over space and time. They

	revealed the lag effects of climatic variables on cholera considering household-level socio-economic factors at the provincial level in Mozambique.<br><br>The study's objective is well-defined, and it employs a valid methodology. Sufficient details were provided to ensure that the method can be replicated. From a public health perspective, the study is highly relevant, given the recent rise in global cholera cases and the need to implement appropriate interventions and preventive measures. We propose that the authors make some revisions and clarifications before the paper can be accepted for publication.<br><br>Minor revisions<br><br>I suggest that the authors consider reorganizing the introduction to improve its clarity. In its current form, the introduction is too lengthy and lacks a well-defined structure. To improve this, the introduction should be condensed and focus on presenting a brief overview of cholera, including known risk factors, followed by a presentation of the number of cases and deaths reported to the WHO worldwide, in Sub-Saharan Africa, and specifically in Mozambique. The introduction should end with a clear statement of the research gap that the authors are addressing and the aim of the study. Currently, the research gap the authors are addressing is not clearly outlined and could benefit from additional clarification.<br><br>Additionally, most of the results in the paper duplicate the information presented in the tables. The authors should consider revising the writing of some of the results presented in the tables. Furthermore, Figure 1 is unclear since variables with different measurement units are compared, and the vertical axes are not labeled.<br><br>Finally, as a general note, it is recommended that the paper be revised to ensure language clarity and correct any grammatical errors, particularly in the introduction, lines 38-41, 42-46, and 57-60.<br><br>Major revisioins<br><br>There appears to be some confusion about the language used in the paper regarding the incidence. Is the incidence being reported as a cumulative incidence, which considers the total number of people at risk as the denominator, or as incidence rates, which consider the study sample’s individual times at risk (person-time)? I believe the paper utilized cumulative incidence and the language ought to reflect that.<br><br>There are no mentions of the study design, the statistical significance level chosen by the authors and whether the study was approved by an institutional review board or ethics committee. Please clarify these omissions.<br><br>It is highly recommended that the authors discuss additional limitations of the study, especially those related to the rigor and accuracy of climatic data, which have been sourced from the National Center for Environmental Prediction. Briefly describe how the data was collected.

VERSION 1 – AUTHOR RESPONSE

COMMENTS FROM REVIEWER 1

Comment 1: The authors report the results of an ecologic study (study design not mentioned) about the effects of climatic and social-economic variables on cholera incidence in Mozambique, in a 19-year period. They observed that temperature, precipitation, relative humidity, a vegatation index, sharing toilet facilities and owning a radio, were associated with the cholera risk.

Response: Thank you for pointing this out. We agree with this comment. Therefore, we would like to confirm that this study is an ecological longitudinal retrospective study. We have thus updated the title of the manuscript as “Spatial temporal analysis of climate and socio-economic conditions on cholera incidence in Mozambique from 2000-2018: an ecological longitudinal retrospective study”.

Comment 2: The statistical methods were properly described and conducted. The discussion brings results from similar studies, with an updated bibliography.

Response: Thank you for the observation.

Comment 3: I would suggest that the authors expand the discussion of the study's limitations.

Response: Thank you for the observation. We have updated the discussion with more details on the study limitations.

Comment 4: My particular concern is the issue of the ecological fallacy. By working with mean values, one would not be obscuring the existence of population groups at greater risk, or exposed to different climatic conditions?

Response: Thank you for the observation. The data was provided at district level, which was already aggregated from the disease surveillance system, but we further aggregated the data to provincial level due to high number of districts reporting zero cholera cases. We acknowledge this in the limitation.

Comment 5: An additional concern is related to the effect of the social-econiomic variables on the cholera risk. Why "owning a radio" is associated with a lower risk, and a mobile phone with its increase?

Response: Thank you for this observation. We reran the model and included the DHS derived SES variables as non-linear terms in the model similar to the climatic factors. With this change, we observed the expected reduction in cholera risk with high proportion of phone ownership. See Table 3 in the revised manuscript.

Comment 6: My final question is related to the cholera time trend in Mozambique. How does the model explain the observed reduction of the incidence over time?

Response: Thank you for this comment. In this analysis we did not explicitly include factors such as interventions (these data are hard to acquire and not widely available) that could explain the reduction in cholera incidence over time, however we allowed the spatio-temporal covariance in the model to vary by year thus capturing the long-term inter-annual changes in incidence. This implicitly captures the unmeasured confounders including interventions that may have been implemented during the study period.

COMMENTS FROM REVIEWER 2

Comment 1: The authors proposed a study to assess how climatic variability and socioeconomic conditions impact cholera incidence in Mozambique, while considering the lagged patterns over space and time. They revealed the lag effects of climatic variables on cholera considering household-level socio-economic factors at the provincial level in Mozambique.

Response: Thank you for the observation.

Comment 2: The study's objective is well-defined, and it employs a valid methodology. Sufficient details were provided to ensure that the method can be replicated. From a public health perspective, the study is highly relevant, given the recent rise in global cholera cases and the need to implement appropriate interventions and preventive measures.

Response: Thank you for the observation.

Comment 3: We propose that the authors make some revisions and clarifications before the paper can be accepted for publication.

Response: Thank you for the observation and we have made revisions and edits based on the suggestions proffered.

Comment 4: I suggest that the authors consider reorganizing the introduction to improve its clarity. In its current form, the introduction is too lengthy and lacks a well-defined structure. To improve this, the introduction should be condensed and focus on presenting a brief overview of cholera, including known risk factors, followed by a presentation of the number of cases and deaths reported to the WHO worldwide, in Sub-Saharan Africa, and specifically in Mozambique. The introduction should end with a clear statement of the research gap that the authors are addressing and the aim of the study. Currently, the research gap the authors are addressing is not clearly outlined and could benefit from additional clarification.

Response: Thank you for the suggestion. We have made revisions and updated the introduction section.

Comment 5: Additionally, most of the results in the paper duplicate the information presented in the tables. The authors should consider revising the writing of some of the results presented in the tables. Furthermore, Figure 1 is unclear since variables with different measurement units are compared, and the vertical axes are not labeled.

Response: Thank you for the suggestion. We have revised and updated the results section of the manuscript. We improved Figure 1 based on the suggestions with clear axis labels. In addition, we provide a new figure in the supplementary, S13 Figure where we plot each covariate in its own panel. If this is preferable we can replace the main figure with that one.

Comment 6: Finally, as a general note, it is recommended that the paper be revised to ensure language clarity and correct any grammatical errors, particularly in the introduction, lines 38-41, 42-46, and 57-60.

Response: Thank you for the observation. We have revised and updated the manuscript based on the suggestion.

Comment 7: There appears to be some confusion about the language used in the paper regarding the incidence. Is the incidence being reported as a cumulative incidence, which considers the total number of people at risk as the denominator, or as incidence rates, which consider the study sample’s individual times at risk (person-time)? I believe the paper utilized cumulative incidence and the language ought to reflect that.

Response: Thank you for the observation. We believe for this study , incidence rate applies since we primarily model monthly incidental cases of cholera. We use population as denominator to compute the rates.

Comment 8: There are no mentions of the study design, the statistical significance level chosen by the authors and whether the study was approved by an institutional review board or ethics committee. Please clarify these omissions.

Response: Thank you for pointing this out.

Regarding the first question, this was an ecological retrospective longitudinal study and we have updated the title and methods section to reflect this.

On significance level, the since the analysis follows a Bayesian framework, we report 95% credible intervals and for the DLNM Relative risk confidence bands are also based on 95% confidence intervals, in essence we use 5% significance level. We have updated the methods to include this.

Regarding ethical approval, we used aggregated secondary data from the Ministry of Health (MoH), for which ethical approval was not required.

Comment 9: It is highly recommended that the authors discuss additional limitations of the study, especially those related to the rigor and accuracy of climatic data, which have been sourced from the

Response: Thank you for the observation. We have updated the limitation section and added a sentence in the methods section backing the validity and reliability of the climatic data used.

In addition to the above comments, all spelling and grammatical errors pointed out by the reviewers have been corrected.

VERSION 2 – REVIEW

**REVIEWER NAME**	Luna, Expedito
**REVIEWER AFFILIATION**	University of Sao Paulo, Instituto de Medicina Tropical
**REVIEWER CONFLICT OF INTEREST**	None.
**DATE REVIEW RETURNED**	19-Jul-2024

**GENERAL COMMENTS**	In my view the authors have properly addressed the issues raised by the reviewers, and the manuscript is sutiable for publication.<br>A minor issue: in pg 8, 2nd line, it reads 2000 - 20024. Please correct it.

**REVIEWER NAME**	Moise, Kenny
**REVIEWER AFFILIATION**	Universite Quisqueya, Laboratoire Santé-Environnement
**REVIEWER CONFLICT OF INTEREST**	No competing interests
**DATE REVIEW RETURNED**	26-Jul-2024

**GENERAL COMMENTS**	We appreciate the authors for incorporating our suggestions and making global improvements to the article. However, the language clarity in the article still requires enhancement, so significant editing is necessary, even to the article's title, before publication. Furthermore, we recommend a statistical review of the methods and results. Besides these comments, kindly ensure that the article is accepted for publication in the journal.
